# New Game-Theoretical Approach to Marketing Cooperatives in Fisheries

**DOI:** 10.1007/s11538-026-01757-6

**Published:** 2026-09-26

**Authors:** Inmaculada López, Manuel Gámez, Ana Belén Castaño-Fernández, József Garay, Zoltán Varga

**Affiliations:** 1 Department of Mathematics, University of Almería, Ctra. de Sacramento S/N, 04120 La Cañada de San Urbano, Almería, Spain; 2 Institute of Evolution, HUN-REN Centre for Ecological Research, Konkoly-Thege M. út 29–33, 1121 Budapest, Hungary; 3 Department of Mathematics and Modelling, Institute of Mathematics and Basic Science, Hungarian University of Agriculture and Life Sciences, Páter K. U. 1, 2100 Gödöllő, Hungary; 4 Center for the Development and Transfer of Mathematical Research to Industry (CDTIME), University of Almería, Ctra. de Sacramento S/N, 04120 La Cañada de San Urbano, Almería, Spain

**Keywords:** Fisheries, Marketing cooperative, Game model, Repeated game

## Abstract

While most game-theoretical models in fisheries deal with the conflict of exploitation of the same resource, unlike these models, the present paper is dedicated to marketing conflicts, always accounting for the underlying population dynamics. In the context of marketing cooperatives in fisheries, three models are combined: the classical fishing effort model based on logistic population dynamics, a game-theoretical model of the conflict between a marketing cooperative and its members, and a Cournot-type oligopoly market model. The latter, in particular, means that the market price is determined by the total supply on the market. The fishers selling fish outside the marketing cooperative are punished. In the present paper, within this complex model, based on Pareto optimality, a particular cooperative solution, the so-called *nearly ideal solution,* is introduced and compared with the previously studied, special non-cooperative solution, also called *attractive solution*. In fact, for the respective repeated games, counting with the underlying fishing effort dynamics, the two solution types are compared. Results show that the cooperative solution yields higher total payoffs if at least one member fishes, one member is unfaithful, and a specific pricing condition holds. Additionally, increasing the punishment parameter stabilizes the cooperative by ensuring members contribute their catch, while higher fishing efforts increase catches without depleting stocks but require stricter penalties to prevent free-market diversion.

## Introduction

The problem of optimization in conflict situations is the typical area of application of game theory (for modeling general conflicts see also Molnár et al. 1988). In the classical case, for the interested parties (players), the different decision options (possible strategies) are determined. Furthermore, the strategy choice of all players (forming a multi-strategy) determines the utility (payoff) of each player. Formally, in this way a *normal form game* is given**.** A solution of such a game would be an appropriate choice of a multi-strategy. According to the approach to this choice, we obtain different solution concepts. A reprint of a classical monograph on game theory and its applications is Von Neumann and Morgenstern (2007), recent, concise and multi-disciplinary introductions to the subject are e.g. Leyton-Brown and Shoham (2022).

In the context of fisheries, most game-theoretical models deal with the conflict of exploitation of the same resource, (see Clark 1990). Examples of modeling water distribution problems with game theory are e.g. Szidarovszky et al. (2013) and Kicsiny et al. (2014a). Unlike these models, the present paper deals with marketing conflicts, where the conflicting players fish from different resources, always accounting for the underlying population dynamics.

A pervasive issue in these marketing conflicts, particularly within primary sector cooperatives, is the opportunistic behavior of members who divert part of their production to unofficial markets. This phenomenon, known in the academic literature as “side-selling”, represents a severe threat to cooperative stability and marketing strategies (Benos et al. 2024). A clear example of this dynamic can be observed in fishing cooperatives, such as those documented on the Baja California Peninsula, where organizations face constant tensions due to fishers selling outside official channels to evade quotas or seek immediate profits (Bórquez et al. 2009). Incorporating this structural condition is essential to properly model the incentive and retention problems addressed in this study.

The starting point of the present study is a basic model of Varga et al. (2010), consisting of three components: a game-theoretical model of the conflict between a marketing cooperative and its members, a Cournot-type oligopoly market model, and the partial adaptive dynamics of Garay (2002), developed for evolutionary game theory, is applied for the dynamic strategy choice of the players. (Some references to this work are e.g. Caristi et al. (2016) and Yuan et al. (2023)). As a further development, in Gámez et al. (2018) the model of Varga et al. (2010) is modified such that the catch is determined by the fishing effort model based on a logistic population dynamics (see e.g. Clark 1990). References to the model of Gámez et al. (2018) are e.g. Abedian et al. (2022) and Peleckis (2021). A recent application of a game model with a distinguished cooperative solution for sustainable groundwater use is Piscopo et al. (2024). Integrating these three components into a single framework is necessary because analyzing them in isolation provides only a partial view: Cournot competition determines market prices based on aggregate supply, population dynamics set the biological limits on that supply to prevent stock depletion, and game theory models individual choices under economic penalties.

Different types of cooperatives can be formed. We have in mind a *marketing cooperative*, the role of which is to help the members sell their product. As a matter of fact, in our case, the single fishers are small entrepreneurs, offering to sell a relatively small quantity of fish. The large supermarket chains wouldn’t buy small quantities of fish, but a cooperative of fishers can offer a larger quantity and negotiate a good price for future catches. In this situation, for the cooperative the cost of buying fish from fishers is recovered from selling the same fish to the supermarket chain at a contracted price. That is why these items are not included in our model.

The paper is organized as follows: In Sect. 2, the basic model and the previously studied, particular non-cooperative solution is recalled. Furthermore, the concept of the cooperative, nearly ideal solution is introduced and illustrated. In Sect. 3, for repeated games we give a comparison of this new, cooperative solution with the previous, non-cooperative solution. It is shown in what sense the nearly ideal solution of a repeated game is better than the equilibrium solution considered earlier in Gámez et al. (2018). Then the effects of the changes in key model parameters such as penalty parameter and fishing effort are studied. Discussion and outlook close the paper.

## Basic Model of a Marketing Cooperative in Fisheries

### Fish Dynamics and Market Model

From Gámez et al. (2018) we recall the following game-theoretical model. Suppose that *n* micro or small fishing companies (for short, also called fishers) fish in independent waters. For a more efficient marketing, they form a cooperative that can negotiate a better price with supermarket chains. The fishing companies, by contract, commit to sell all their catch to the cooperative at unit price *p*. Let us assume that each company *i* fishes during a time interval [0,T], at the end of which they sell the catch

1 $$L_{i} = \int_{0}^{T} {E_{i} } \rho_{i} z_{i} (t){\mkern 1mu} {\mathrm{d}}t,$$

where ${E}_{i}$ is the fishing effort, ${\rho}_{i}$ the catchability, and ${z}_{i}(t)\in [0,T]$ the solution of the differential equation

2 $$\dot{z}_{i} = r_{i} z_{i} \left( {1 - \frac{{z_{i} }}{{K_{i} }}} \right) - E_{i} \rho_{i} z_{i} \quad (i = 1,2, \ldots ,n)$$

with given initial value ${z}_{i}(0)$. Here ${r}_{i}$ is the Malthus parameter, and ${K}_{i}$ the carrying capacity of the *i*-th fishing area. We note that if the fishing effort is not too high, i.e. ${E}_{i}<\frac{{r}_{i}}{{\rho}_{i}},$ then dynamics (2) has a unique positive equilibrium.

$$z_{i}^{*} = K_{i} \left( {1 - \frac{{E_{i} \rho_{i} }}{{r_{i} }}} \right) > 0\;\;{\mathrm{with}}\;\;0 < z_{i}^{*} < K_{i} .$$

The solution of Eq. (2) with initial value ${z}_{i}\left(0\right)$ is

$$z_{i} \left( t \right) = \frac{{\frac{{z_{i}^{*} \cdot z_{i} \left( 0 \right)}}{{z_{i}^{*} - z_{i} \left( 0 \right)}}}}{{\frac{{z_{i} \left( 0 \right)}}{{z_{i}^{*} - z_{i} \left( 0 \right)}} + e^{{ - \left( {r_{i} - E_{i} \rho_{i} } \right)t}} }}\;(t \ge 0),$$

and hence from (1), by the time *T* of selling, the total catch is

3 $$L_{i} = \frac{{ - E_{i} \rho_{i} z_{i}^{*} }}{{r_{i} - E_{i} \rho_{i} }}ln\frac{{e^{{ - \left( {r_{i} - E_{i} \rho_{i} } \right)T}} z_{i}^{*} }}{{z_{i} \left( 0 \right) + e^{{ - \left( {r_{i} - E_{i} \rho_{i} } \right)T}} \left( {z_{i}^{*} - z_{i} \left( 0 \right)} \right)}}\;\;\;\;\;\left( {i = {1},{2}, \ldots ,n} \right)$$

Now the conflict between the cooperative and its members consists in the following: Although each member is committed to sell all its catch ${L}_{i}$ to the cooperative at the contracted price *p*, it may occur that at time *T*, the price on the free market is higher than the contracted price. Then some “unfaithful” members may decide to sell only a part ${x}_{i}\in [\mathrm{0,1}]$ to the cooperative, selling the remaining part ${1-x}_{i}\in [\mathrm{0,1}]$ on the free market. Suppose that the unfaithful members, although fishing in different waters, will sell on the same market of the region. For simplicity, it is reasonable to suppose that the market price *q* can be obtained from a Cournot-type oligopoly model with joint linear price function: With appropriate constants and $x=({x}_{1},{x}_{2},...,{x}_{n})$ we have

$$q(x): = a - b\sum\limits_{i = 1}^{n} {L_{i} } (1 - x_{i} ).$$

(We note that *a* can be interpreted as the market price in the absence of supply, say the import price.) Of course the cooperative members will be motivated to sell in the market, rather than to the cooperative, only if q(x)>p. Therefore, the latter will be assumed throughout the paper.

### Normal Form Game

The conflict between the cooperative and its members consists in the following: If at the moment of commercialization of the catch, the market price is higher than the contracted price, then the members may have a tendency to sell at least a part of their catch in the free market, but the cooperative can punish them for “infidelity”. As in the previous section, suppose that the *i*-th member of the cooperative, i.e. *Player i* (*i*=1,2,…,*n*) sells only a part ${x}_{i}\in [\mathrm{0,1}]$ of its catch to the cooperative, selling the remaining part ${1-x}_{i}\in [\mathrm{0,1}]$ on the free market. ${x}_{i}$ is considered the strategy of Player *i.* The strategy of the cooperative, *Player n*+1, will be $y\in [\mathrm{0,1}]$, expressing the intensity of punishment. The elements

$$(x,y)=({x}_{1},{x}_{2},...,{x}_{n},y)\in [\mathrm{0,1}{]}^{n}\times [\mathrm{0,1}]$$

are called *multi-strategies*.

Let c > 0 be the production cost of a unit biomass, and $\beta \ge 1$ a punishment parameter. Then for each $i \in \overline{1,n}$, the payoff of Player *i* is its net income – punishment:

4 $$\begin{gathered} f_{i} \left( {x,y} \right): = \left[ {\left( {p - c} \right)x_{i} L_{i} + \left( {q\left( x \right) - c} \right)\left( {1 - x_{i} } \right)L_{i} - \beta y\left( {q\left( x \right) - p} \right)\left( {1 - x_{i} } \right)L_{i} } \right]\; \hfill \\ = L_{i} \left\{ {\left( {p - c} \right)x_{i} + \left( {1 - x_{i} } \right)\left[ {q\left( x \right) - c - \beta y\left( {q\left( x \right) - p} \right)} \right]} \right\}. \hfill \\ \end{gathered}$$

For Player *n*+1, the payoff is the total punishment collected:

5 $$f_{n + 1} \left( {x,y} \right): = \beta y\left( {q\left( x \right) - p} \right)\mathop \sum \limits_{j = 1}^{n} L_{j} \left( {1 - x_{j} } \right)\;.$$

From definitions (4) and (5), with notation $f:=({f}_{1},{f}_{2},...,{f}_{n+1})$*,* we have a normal form game

6 $$\Gamma : = \left( {\left[ {0,1} \right]^{n} \times \left[ {0,1} \right],f} \right).$$

Next, from Gámez et al. (2018) we adapt a special non-cooperative solution of game Γ, see also the references therein.

### A Non-Cooperative, Equilibrium Solution Concept for Multi-Player Games With a Distinguished Player

For games of the form (6), we introduce a special equilibrium solution with the following property: if Player *n*+1 sticks to its equilibrium strategy, the other players cannot increase their payoff even if they deviate together from their equilibrium strategies. In our model the cooperative is the distinguished player. The formal definition is the following:

#### Definition 4

Multi-strategy $(x^{0}, y^{0} )$ is said to be an *attractive solution* of the normal form game Γ, if the following conditions are satisfied:

(A) $$f_{j} (x,y^{0} ) \le f_{j} (x^{0} ,y^{0} ),\;(j \in \overline{1,n} ,x \in [0,1]^{n} );$$
(B) $$f_{n + 1} (x^{0} ,y) \le f_{n + 1} (x^{0} ,y^{0} )\;\;\left( {y \in [0,1]} \right).$$

We note that by special choices of $x \in [0,1]^{n}$ in inequalities (A), together with condition (B) we obtain that an attractive solution is a particular Nash equilibrium, and hence it is considered a special *non-cooperative solution*.

### Cooperative Solution of the Same Game, Based on Pareto Optimality

For the definition of a special cooperative solution of a (6) type game, first we recall the concept of Pareto optimality (also called Pareto efficiency). Considering the multi-criterial (or vector) optimization problem

7 $$f\left( {x,y} \right) \to max$$

$$s = \left( {x,y} \right) \in [0,1]^{n} \times \left[ {0,1} \right] = :G,$$

multi-strategy $({x}^{*},{y}^{*})\epsilon G$ is said to be *Pareto optimal for* f (and $f\left({\mathrm{s}}^{*}\right)$ is called *Pareto optimal value*)${\mathrm{s}}^{*}$, if there is no s∈G with $f_{i} \left( {{\mathrm{s}}^{*} } \right) \le f_{i} \left( s \right)\;\;\;\;\;(i \in \overline{1,n + 1} )$, where at least one inequality is strict.

The set *P* of Pareto optimal values is called the *Pareto frontier* of *f*:

$$P: = \left\{ {f\left. {\left( s \right)} \right|s \in G{\text{ is Pareto optimal for }}f } \right\}.$$

#### Definition 5

The elements of the counter image of *P* under the function *f* are called *cooperative solutions of game* (6). In other words, a multi-strategy is a cooperative solution if there is no other multi-strategy choice that makes one player better off without making some other player worse off.

**Fig. 1:**
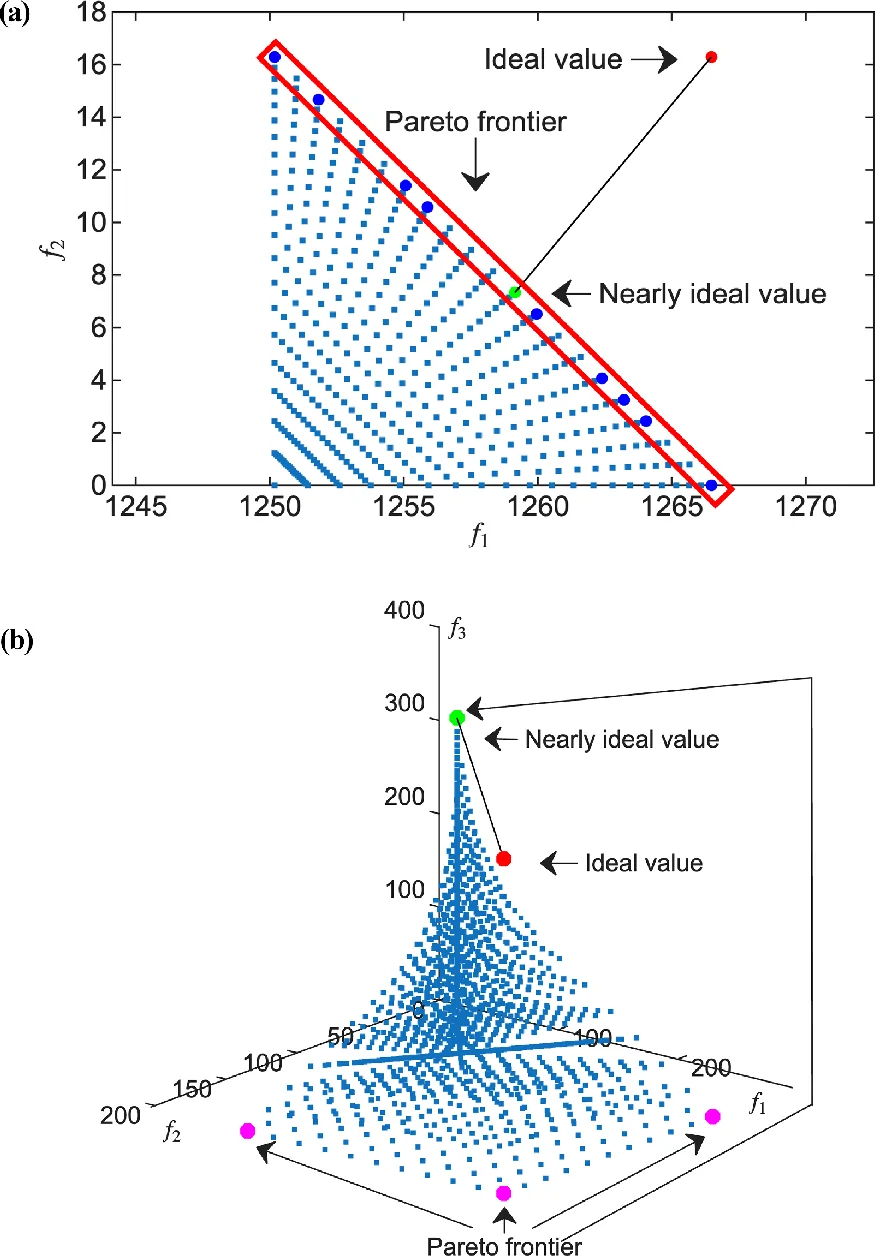
**a** The payoffs (total net revenues; *b*=0.000035; *r*=0.25; E=4.5;ρ=0.05;*T*=14; *K*=1485; *z*(0)=161.77, nearly ideal solution. **b** The total revenues (payoffs) $({f}_{1},{f}_{2},{f}_{3})$ for two Fishers and the cooperative with parameters *p*=1.5;c=(3/4)p;β=1;*a*=2.5;b=0.000035; *r*=[0.25,0.21]; *E*=4[2,1];$\rho =[\mathrm{0.01,0.015}]$;*T*=14; *K*=[1100,1000]; *z*(0)=[450,450], nearly ideal solution (Color figure online)

A straightforward check shows that cooperative solutions of game (6) can be obtained by solving the scalarizations of the vector optimization problem (7), by weighting the single functions${f}_{i}$: Indeed, let ${relint\Delta }_{n+1}$ be the *relative interior of the standard simplex* in **R**^n+1^, i.e. the set of positive vectors $\lambda =\left({\lambda}_{1},...,{\lambda}_{n+1}\right)$ with components summing to 1. Then for every${\lambda \in relint\Delta }_{n+1}$, any solution of the scalar optimization problem

8 $$f_{\lambda } \left( s \right): = \mathop \sum \limits_{i = 1}^{n + 1} \lambda_{i} f_{i} \left( s \right) \to max$$

s∈G

is *Pareto optimal* multi-strategy for the vector-valued function $f=({f}_{1},{f}_{2},...,{f}_{n+1})$, and hence it is also a cooperative solution of the normal form game (6), see Geoffrion (1968). In the latter reference it is also shown that, except for some degenerate cases, in this way a description of the whole Pareto frontier *P* can be obtained, as it will be shown in Figs. 1a and b of the next subsection. In principle, by solving the scalarized optimization problems (8), and letting run $\lambda \in {relint\Delta }_{n+1}$, in general we obtain an infinite set of cooperative solutions. This makes it possible to pick one of them according to a further requirement. This is done in the next subsection.

### Nearly Ideal Solution of the Game

Based on an original idea by Salukvadze (1971a, 1971b), from Gámez et al. (2018) we recall the definition of nearly ideal value for game (6), see also Kicsiny et al. (2014a) and Piscopo et al. (2024). First, for every $i \in \overline{1,n + 1}$,

9 $$\omega_{i} : = \mathop {\sup }\limits_{s \in G} f_{i} \left( s \right) { }$$

is called the *ideal value for player i,* and vector

$${\Omega }: = \left( {\omega_{1} ,\omega_{2} ,, \ldots ,\omega_{n} ,\omega_{n + 1} } \right)$$

is said to be the *ideal value for game *Γ*.*

Obviously, in general, there is no multi-strategy s∈G such that f(s)=Ω. However, in the Pareto frontier there may be a point nearest to the ideal value.

#### Definition 6

Multi-strategy ${s}^{0}\in G$ is called a *nearly ideal solution of game* (6), if

10 $$\left| {\Omega - f\left( {s^{0} } \right)} \right| = \mathop {inf}\limits_{p \in P} \left| {\Omega - p} \right|.$$

The intuitive interpretation of the nearly ideal solution of a game is the following: As we have seen in Definition 5, the elements of the Pareto frontier provide a variety of cooperative solutions of the game. The concept of nearly ideal solution provides a reasonable way to select a unique cooperative solution, acceptable for all players.

$f\left({s}^{0}\right)$ is called the *nearly ideal value of the game*.

In the numerical realization of the concrete models it will be seen that the supremums in (9) and the infimum in (10) are reached.

Next we illustrate the above notions, providing examples with minimal dimensions, geometrically representable in the plane.

## Analytical Results

In reality, the fishing and marketing activity is usually repeated several times. Let us suppose that our fishery game (6) is played *M* times. The fishery game (6) of the section is played in a time interval [0,*T*]. Assume that fishing and selling are played *M* times, in consecutive intervals [0,*T*], [*T*,2* T*],…,[(*M*-1)*T*, *MT*]. *M* represents the frequency with which fishermen go out to sea over the total duration *T*, serving as a partition of the interval. Then for every $m \in \overline{0,M - 1}$, we define a normal form game ${\Gamma}_{m}$, where the set of multi-strategies is always the same $[\mathrm{0,1}{]}^{n}\times [\mathrm{0,1}]$, but the payoff function ${f}_{i, m}$ of player *i* in the *m*-th game, in analogy with definitions (4) and (5), is defined as follows:

11 $$\begin{gathered} f_{i, m } \left( {x,y} \right): = \left[ {\left( {p - c} \right)x_{i} L_{i, m } + \left( {q\left( x \right) - c} \right)\left( {1 - x_{i} } \right)L_{i, m } - \beta y\left( {q\left( x \right) - p} \right)\left( {1 - x_{i} } \right)L_{i, m } } \right]\;\; \hfill \\ = L_{i, m } \left\{ {\left( {p - c} \right)x_{i} + \left( {1 - x_{i} } \right)\left[ {q\left( x \right) - c - \beta y\left( {q\left( x \right) - p} \right)} \right]} \right\}\;\;\;(i \in \overline{{1,n}} )\;, \hfill \\ \end{gathered}$$

and for player (n+1) the payoff is

12 $$f_{n + 1, m } \left( {x,y} \right): = \beta y\left( {q\left( x \right) - p} \right)\mathop \sum \limits_{j = 1}^{n} L_{j, m } \left( {1 - x_{j} } \right).$$

In definitions (11) and (12), ${L}_{i, m}$ is the catch by the *i* -th fishing company during time interval [*m*, (*m*+1)*T*],

$$L_{i, m } : = \mathop \smallint \limits_{mT}^{{\left( {m + 1} \right)T}} E_{i} \rho_{i} z_{i,m} \left( t \right)dt\;\;\left( {m \in \overline{0,M - 1} } \right),$$

where ${z}_{i,m}\left(t\right)$ (t∈[mT,(m+1)T]) is the solution of the underlying population dynamics (fishing effort model) (2) of subSect. 2.1, such that the initial value of ${z}_{i,m+1}$ at *t*=(*m*+1)*T* is the final value of ${z}_{i,m}$ at *t* = (*m*+1)*T*, for $m \in \overline{0,M - 1}$.

In this way, we obtain a finite sequence of normal form games

13 $$\Gamma_{m} : = \left( {\left[ {0,1} \right]^{n} \times \left[ {0,1} \right],f} \right).\;\;\;\;\;\;(m \in \overline{0,M - 1} ).$$

### Attractive Solution of the Repeated Game

Denoting ${\mathbf{1}}: = (1,1, \ldots ,1) \in {\mathbf{R}}^{n}$, for every β>1, $m \in \overline{0,M - 1}$ and multi-strategy $(x,y) \in [0,1]^{n} \times [0,1]$, we obviously obtain

14 $$f_{i, m } \left( {x,1} \right) - f_{i, m } \left( {\mathbf{1},1} \right) = L_{i, m } \left( {1 - \beta } \right)\left( {1 - x_{i} } \right)\left( {q\left( x \right) - p} \right) \le 0 \left( {i \in \overline{1,n} } \right),$$

and

15 $$\begin{gathered} f_{n + 1, m } \left( {\mathbf{1},y} \right) - f_{n + 1, m } \left( {\mathbf{1},1} \right) = \beta y\left( {q\left( \mathbf{1} \right) - p} \right)\mathop \sum \limits_{j = 1}^{n} L_{j, m } \left( {1 - 1} \right) \hfill \\ - \beta \left( {q\left( \mathbf{1} \right) - p} \right)\mathop \sum \limits_{j = 1}^{n} L_{j, m } \left( {1 - 1} \right) = 0. \hfill \\ \end{gathered}$$

Furthermore, it is easy to see that under the conditions a,b>0, a>p and $q\left(x\right)>p$, in case β>1, $x \in [0,1]^{n}$ with $x_{i} < 1$ for some $i \in \overline{1,n}$, inequality (14) is strict.

Hence we obtain the following theorem.

#### Theorem 1

Given M, the number of games played, for any m=0,1,⋯,M-1, multi-strategy $\left(\mathbf{1},1\right)$ is an attractive solution of game ${\Gamma}_{m}$ described in (13).

### Comparison of the Nearly Ideal Solution and the Nash Equilibrium in the Repeated Game

Next, in case of the repeated game, we give some sufficient conditions for a comparison of the total net revenue, i.e. the sum of payoffs at the nearly ideal solution and at the Nash equilibrium:

#### Theorem 2

Let ${s}^{*,m}=\left({x}^{*,m},{y}^{*,m}\right)$ be the nearly ideal solution of game ${\Gamma}_{m}$ defined in (13), and (1;1) an attractive solution of game ${\Gamma}_{m}$. Suppose that the following conditions hold:

(i) There is at least one i-player with ${L}_{i, m }>0,$ that is, there is at least one player who has a non-zero catch.
(ii) At least for one *i*=1,…,*n*, we have ${x}_{i}^{0}<1$, that is, there is at least one member of the cooperative that is “unfaithful” to the cooperative
(iii) $q({x}^{*,m})>p$ (this condition is fulfilled as a hypothesis throughout the paper, due to q(x)>p) for any strategy x.

Then the total net revenue (sum of payoffs) of the players of the game in the nearly ideal solution is always greater than the total net revenue in the Nash equilibrium $\left(1,1\right)$.

#### Proof

The total net revenue of the players of game ${\Gamma}_{m}$ in the nearly ideal solution ${s}^{*,m}$ is the following sum:

$$\mathop \sum \limits_{i = 1}^{n} f_{i,m} \left( {x^{*,m} ,y^{*,m} } \right) + f_{n + 1,m} \left( {x^{*,m} ,y^{*,m} } \right).$$

The total net revenue of the players of game ${\Gamma}_{m}$ in the Nash equilibrium $\left(\mathbf{1},1\right)$ is

$$\mathop \sum \limits_{i = 1}^{n} f_{i,m} \left( {\mathbf{1},1} \right) + f_{n + 1,m} \left( {\mathbf{1},1} \right).$$

Then bearing in mind that conditions (i), (ii) and (iii) hold, we have

$$\begin{gathered} \mathop \sum \limits_{i = 1}^{n} f_{i,m} \left( {x^{*,m} ,y^{*,m} } \right) + f_{n + 1,m} \left( {x^{*,m} ,y^{*,m} } \right) - \left( {\mathop \sum \limits_{i = 1}^{n} f_{i,m} \left( {\mathbf{1},1} \right) + f_{n + 1,m} \left( {\mathbf{1},1} \right)} \right) \hfill \\ \quad = \mathop \sum \limits_{i = 1}^{n} \left\{ {L_{i,m} \left[ {\left( {p - c} \right)x_{i}^{*,m} + \left( {1 - x_{i}^{*,m} } \right)\left( {q\left( {x_{i}^{*,m} } \right) - c - \beta y^{*,m} \left( {q\left( {x_{i}^{*,m} } \right) - p} \right)} \right)} \right]} \right\} \hfill \\ \qquad + \beta y^{*,m} \left( {q\left( {x_{i}^{*,m} } \right) - p} \right)\mathop \sum \limits_{j = 1}^{n} L_{j,m} \left( {p - c} \right) - \mathop \sum \limits_{i = 1}^{n} L_{i,m} \left( {p - c} \right) \hfill \\ \quad = \mathop \sum \limits_{i = 1}^{n} \left\{ {L_{i,m} \left[ {\left( {p - c} \right)x_{i}^{*,m} + \left( {1 - x_{i}^{*,m} } \right)\left( {q\left( {x_{i}^{*,m} } \right) - c} \right)} \right] - L_{i,m} \left( {p - c} \right)} \right\} \hfill \\ \quad = \mathop \sum \limits_{i = 1}^{n} \left\{ {L_{i,m} \left( {p - c} \right)\left( {x_{i}^{*,m} - 1} \right) + L_{i,m} \left( {1 - x_{i}^{*,m} } \right)\left( {q\left( {x_{i}^{*,m} } \right) - c} \right)} \right\} \hfill \\ \quad = \mathop \sum \limits_{i = 1}^{n} L_{i,m} \left( {1 - x_{i}^{{{*},m}} } \right)\left( {q\left( {x_{i}^{{{*},m}} } \right) - p} \right) > 0. \hfill \\ \end{gathered}$$

Note that while, analytically, the failure to satisfy condition (i) or condition (ii) results in both total net revenues being equal (i.e., the strict inequality collapses into an equality), these conditions are structurally necessary from a practical perspective; without them, the cooperative loses its operational purpose and the underlying real-world problem of marketing conflict and opportunistic behavior loses its analytical meaning. Section 4.4 provides an illustrative example showcasing the game's outcomes when some of these theoretical conditions fail.

## Numerical Examples

### Dominance of the Nearly Ideal Solution Over the Nash Equilibrium

#### Example 1

**Fig. 2:**
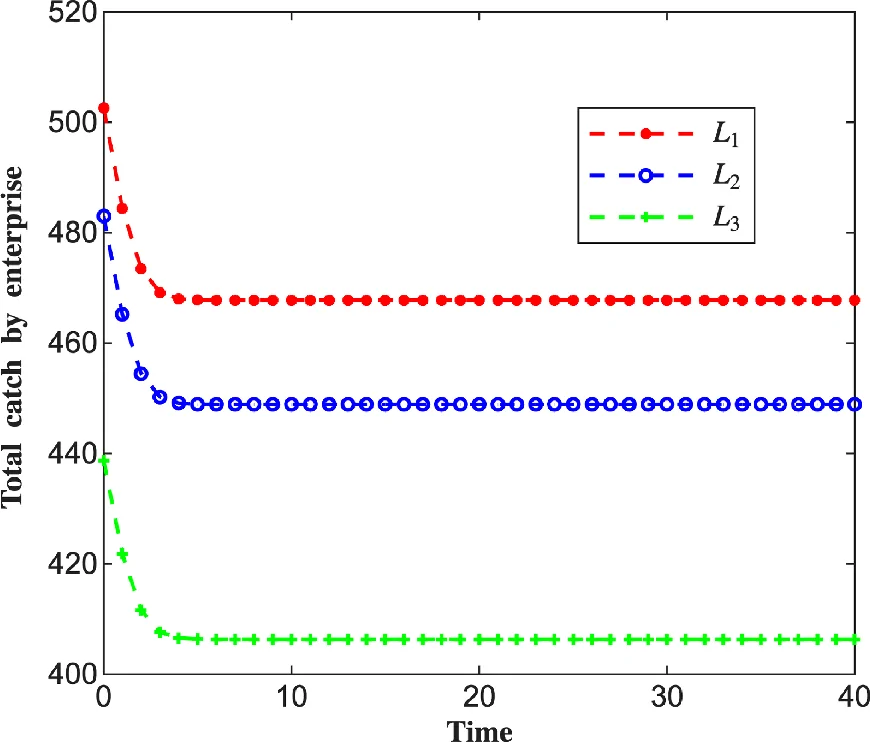
Total catch for each fisher based on the data from Example 1, where ${L}_{i}>0,$ for $i=\mathrm{1,2},3.$ Every fisher records a catch (Color figure online)

**Fig. 3:**
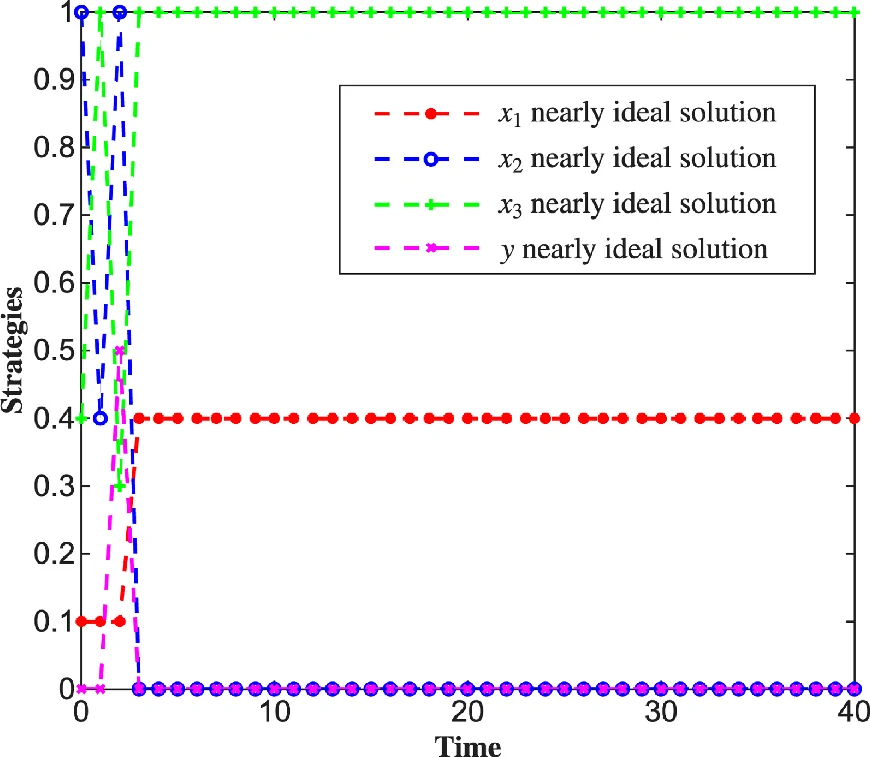
Strategy dynamics corresponding to Example 1, where at least one fisher (in this case, $${x}_{1}$$ and $${x}_{2})$$ is “unfaithful” to the cooperative (Color figure online)

**Fig. 4:**
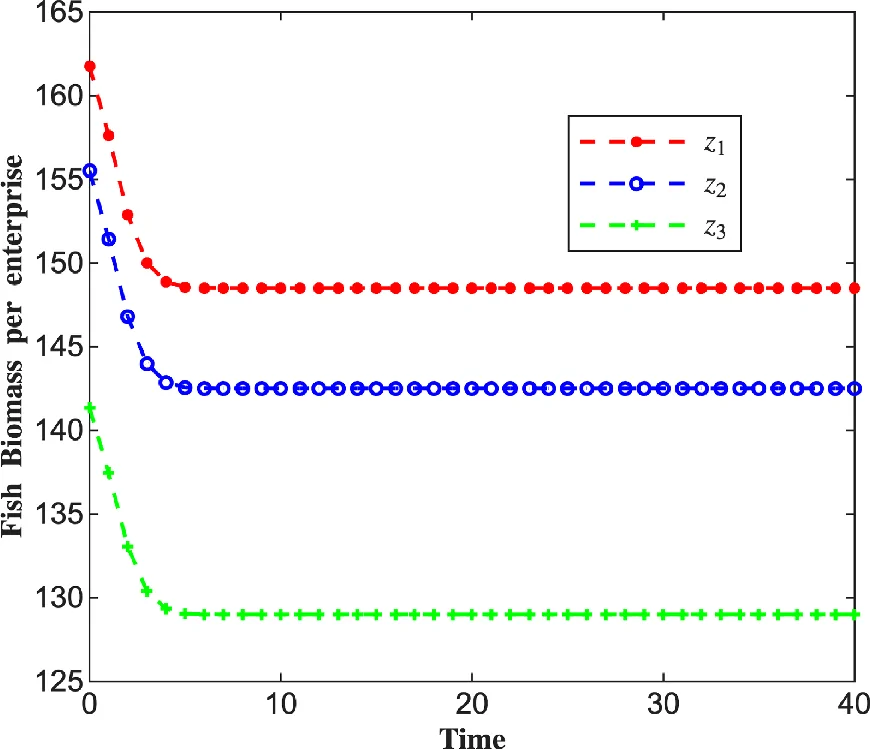
Evolution of fish dynamics (2) corresponding to Example 1, under the condition $q\left({x}^{*,m}\right)>p.$ (Color figure online)

**Fig. 5:**
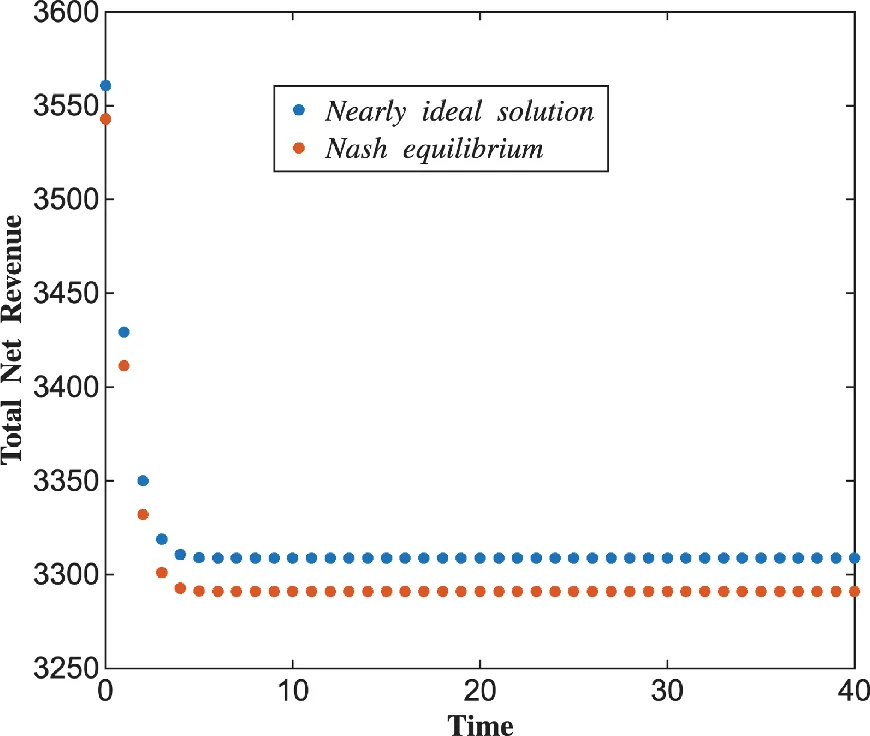
In Example 1, the total net revenue in the nearly ideal solution is always greater than in the Nash equilibrium $\left(1,1\right)$ of the repeated game (Color figure online)

For illustrations of Theorem 2, we considered a marketing cooperative composed of three small enterprises that fish the same species in three different lakes, according to the fishing model showed in (11)–(12), where the parameters are ${K}_{1}=1485;{ K}_{2}=1425;{ K}_{3}=1290;{r}_{i}=0.25; {\rho}_{i}=0.05;{E}_{i}=4.5,$ with $i=\mathrm{1,2},3.$ Additionally, the parameters of the fishing game defined in (13) are $\beta =1;p=9.95;a=10;b=0.000035,c=\frac{3}{4}p;T=14$ days. In Figs. 2, 3 and 4 we illustrate the conditions of Theorem 2. Figure 2 represents the first condition, where at least one enterprise engages in fishing. In this case, all the fishing players obtain a catch. The second condition is given in Fig. 3, where initially players ${x}_{1}$ and ${x}_{2}$ do not sell their entire catch to the cooperative, whereas ${x}_{3}$ does. Finally, using this initial condition set z (0)=(161.77,155.51,141.35), we verify that $q({x}^{*,m})>p$ (third condition) holds. Figure 4 confirms that fishing activity can be sustained across all three lakes. Then, the statement of Theorem 2 is illustrated in Fig. 5. The corresponding development of strategy and catch dynamics is presented in Fig. 3.

### Stabilization of the Cooperative by Setting the Punishment Parameter

In this subsection we show how the cooperative can control the fishers, forcing them to sell at least a part of their catch to the cooperative, contributing in this way to the stabilization of the cooperative itself. (Certain level of the total catch sold to the cooperative may be necessary to justify the existence of the latter.)

#### Example 2

**Fig. 6:**
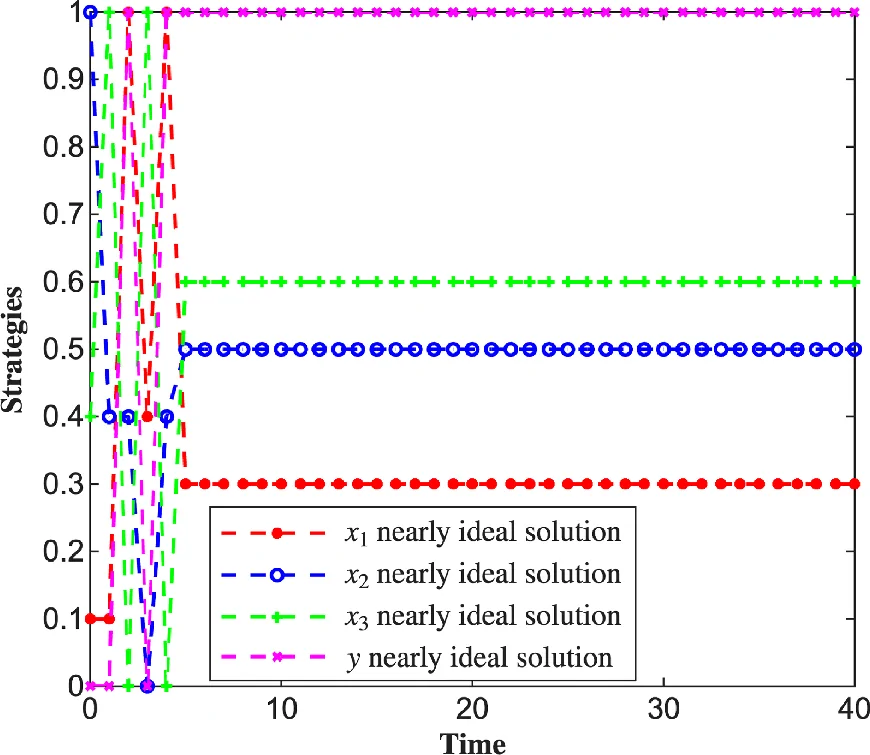
Strategy dynamics for Example 2, in which all fishers sell a part of their catch to the cooperative (Color figure online)

**Fig. 7:**
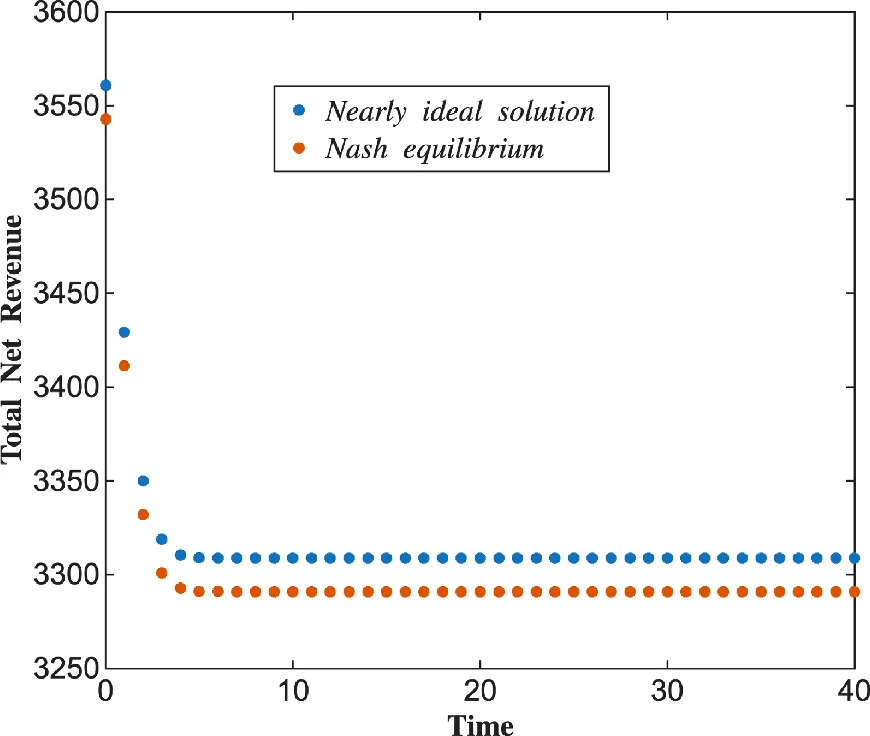
Comparison between solutions from game (13) and the Nash equilibrium corresponding to Example 2. The total net revenue in the nearly ideal solution is greater than the Nash equilibrium $\left(1,1\right)$ (Color figure online)

To assess the penalty effectiveness, we consider the same parameter configuration of Example 1, maintaining β=1. As shown in Fig. 3, the imposed penalty is insufficient to ensure that all fisheries allocate at least part of their catch to the cooperative. In fact, player ${x}_{2}$ sells its entire catch in the free market. If we increase the penalty, setting β=1.4 (while keeping all other parameters unchanged), then the previous issue is resolved. Figure 6 demonstrates how the adjusted penalty stabilizes the cooperative, guaranteeing that all fishers contribute a portion of their catch to the cooperative. However, although this adjustment improves the situation of ${x}_{2}$ within the game, it negatively affects the cooperative selling percentages of the remaining players. Nevertheless, these changes remain within acceptable bounds, i.e. the changed parameters still satisfy conditions of Theorem 2 (see Fig. 7).

**Fig. 8:**
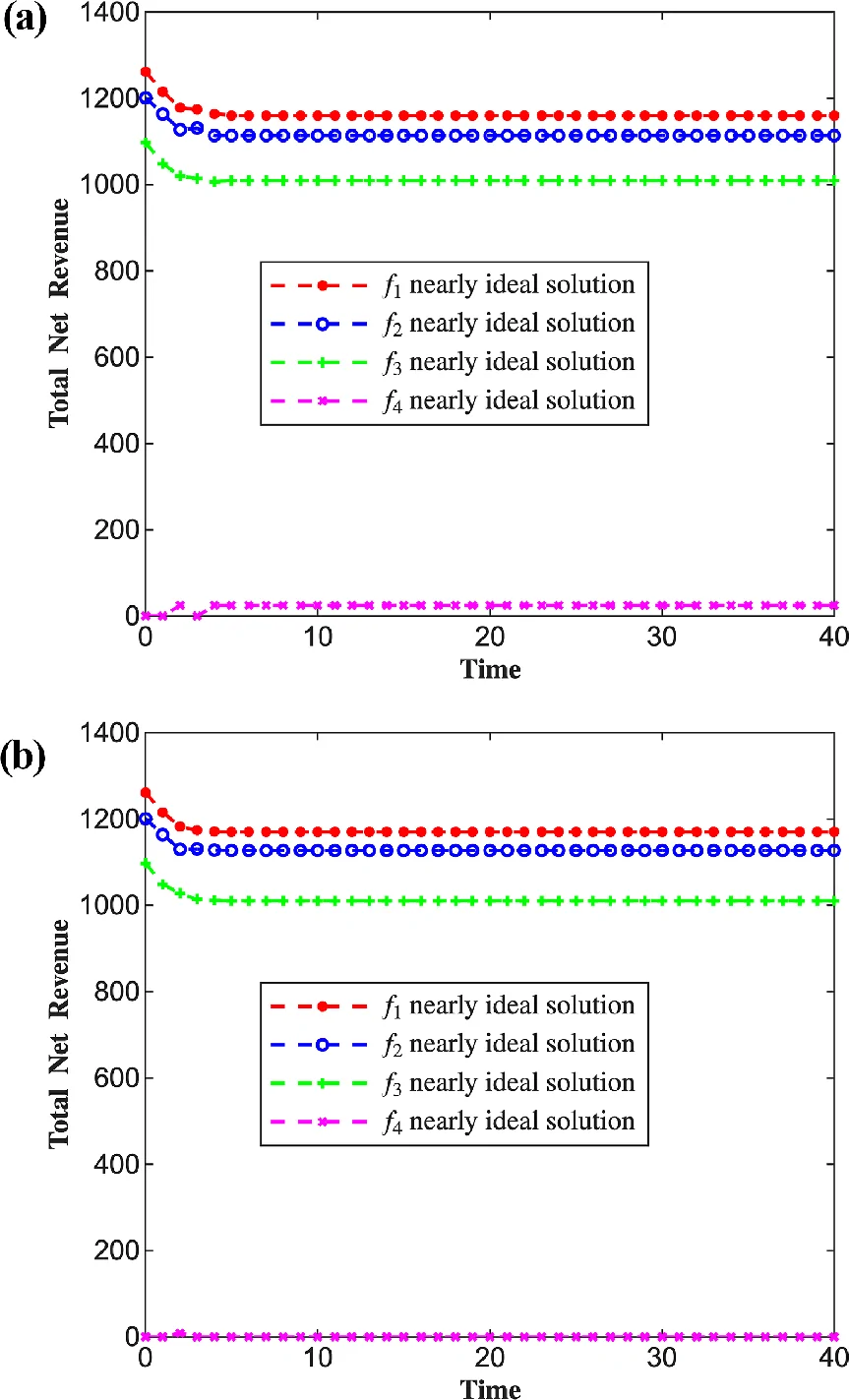
**a** Total net revenue dynamics (11)–(12) for Example 2, regarding the fishers and cooperative, respectively. The cooperative yields benefit when β=1.4. **b** Total net revenue dynamics (11)–(12) for Example 1. In this case, the cooperative’s benefit is zero when β=1 (Color figure online)

Next, Fig. 8a illustrates how the cooperative generates income even when β=1.4 (Example 2) despite the cooperative’s strategy being consistently zero when β=1 (Example 1), as shown in Fig. 8b.

### Effect of Changing Fishing Effort

Fishing effort is a basic quantity underlying the connection between the corresponding population dynamics and the economic activity in fisheries. In fact, fishing effort is a fundamental parameter in dynamic fishing models, as it represents the intensity with which fishers exploit the fish stock. Through the population dynamics model (2), fishing effort ${E}_{i}$, according to (1) determines the catch ${L}_{i}$ during considered time interval [0,T], to be sold by fishing company *i* either to the cooperative or on the free market. The effect of a change in fishing effort is illustrated in the following Example 3.

#### Example 3

**Fig. 9:**
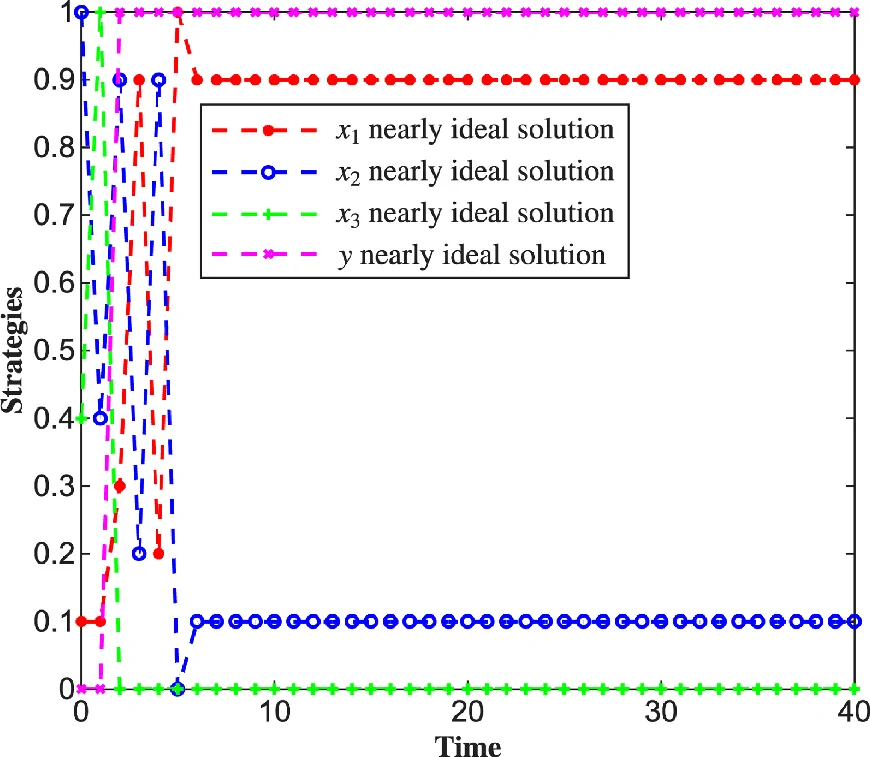
Strategy dynamics for Example 3, where all fishers continue to sell their catch to the cooperative after increasing fishing effort of third fisher to ${E}_{3}=4.7$ (Color figure online)

**Fig. 10:**
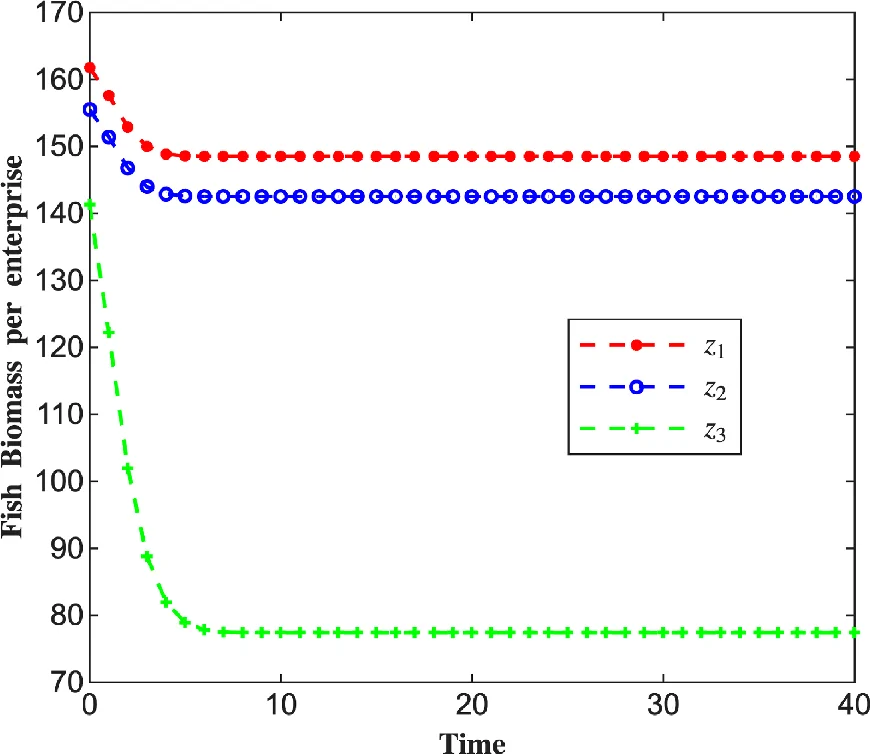
Development of fish dynamics (2) for Example 3. Despite the reduction in the lake’s population, the increased catch levels still ensure sufficient stock for subsequent fishing periods (Color figure online)

**Fig. 11:**
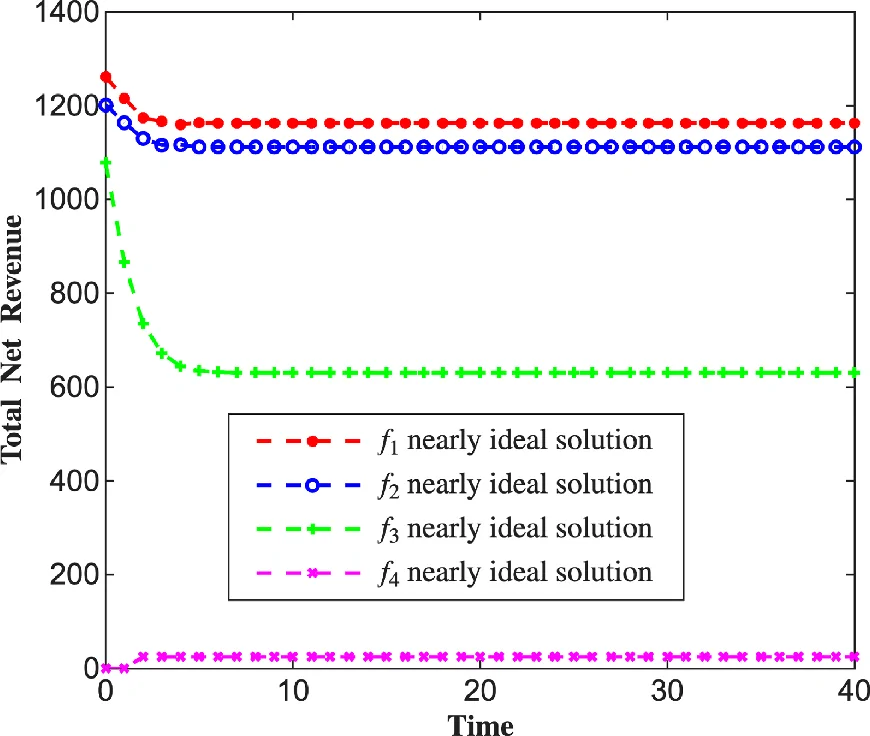
Profit dynamics (11)–(12) for Example 3 regarding the fishers and cooperative, respectively. The cooperative yields benefit after increasing fishing effort of third fisher to ${E}_{3}=4.7$ (Color figure online)

Starting from basic parameter configuration of Example 2, we set ${E}_{3}=4.7$. Under these conditions, and without applying any penalty, the third player sells its entire catch in the free market, leaving no contribution to the cooperative, as shown in Fig. 9. Furthermore, despite the reduced fish population in the lake, increasing the catch still ensures sufficient stock for subsequent fishing periods (see Fig. 10). As in Example 2, the cooperative continues to generate income, as illustrated in Fig. 11.

**Fig. 12:**
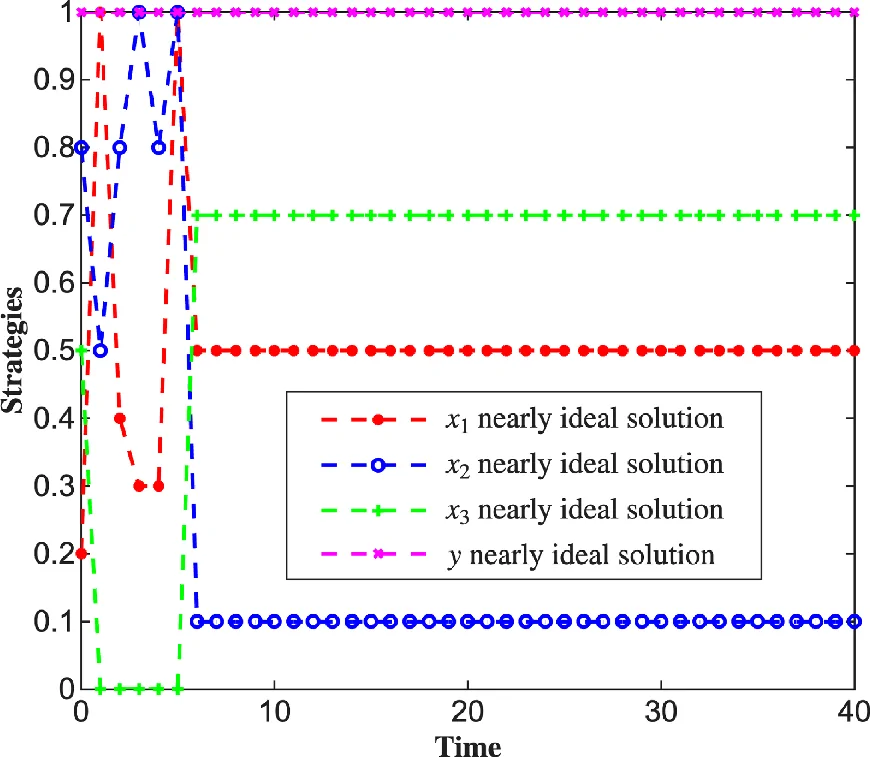
Strategy dynamics for Example 3: all fishers maintain their commitment to the cooperative following an increase in the penalty to β=10 (Color figure online)

**Fig. 13:**
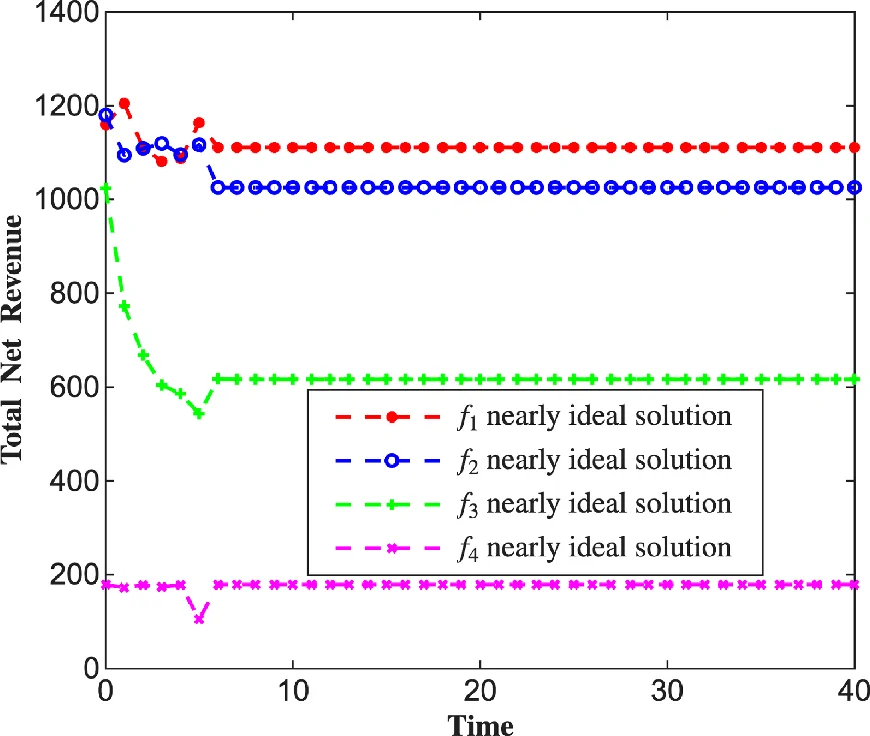
Profit dynamics (11)–(12) in Example 3: the Cooperative shows an increase in benefit following the adjustment of the third fisher’s effort to ${E}_{3}=4.7$ and the penalty to β=10 (Color figure online)

**Fig. 14:**
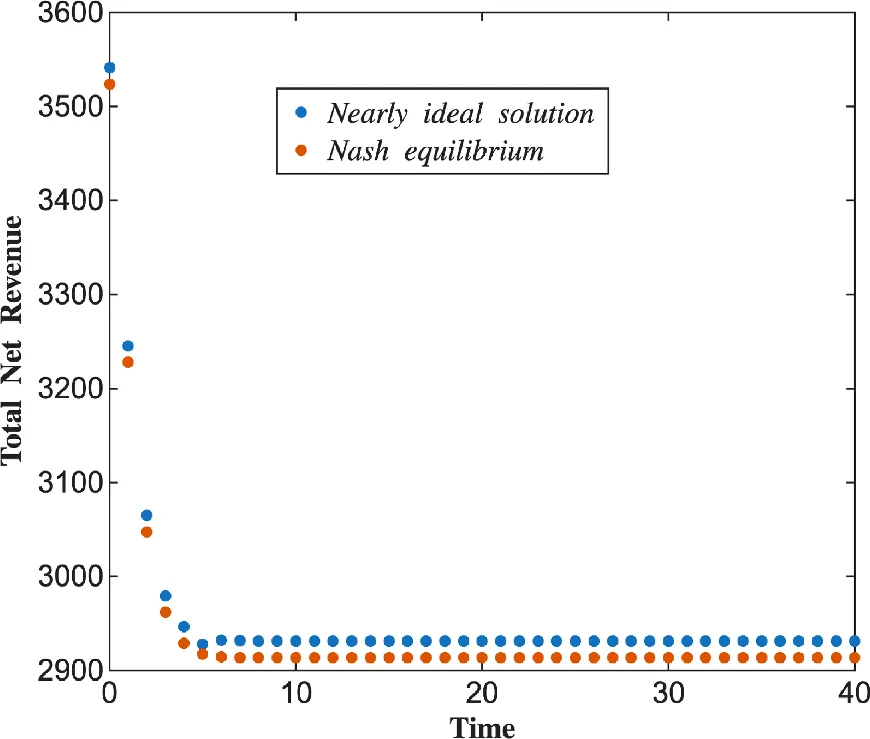
Comparison of total net revenue in Example 3. The nearly ideal solution exceeds the revenue obtained at the Nash equilibrium $\left(1,1\right)$ (Color figure online)

Next, we applied the penalty mechanism to encourage the third fisher to allocate part of its catch to the cooperative. For this purpose, we increased β=10 (maintaining all other parameters, including ${E}_{3}=4.7$). Figure 12 depicts the new dynamic strategies, where all the fishers sell a portion of their catch to the cooperative, as expected. Consequently, the cooperative’s revenues increase due to the stronger penalty (see Fig. 13). Finally, Theorem 2 is valid once again, as demonstrated in Fig. 14.

It is worth noting that while the trajectories exhibit minor non-monotonic fluctuations during the early transient phase (e.g., around time 6), the fundamental result holds: the nearly ideal solution strictly dominates the Nash equilibrium throughout the time horizon.

### Absence of Improvement Over the Nash Equilibrium When the Conditions of Theorem 2 Fail.

While an exhaustive analysis of all scenarios outside the scope of Theorem 2 is beyond the objectives of this paper, this section presents an illustrative case to show how when the theoretical conditions are not satisfied, the nearly ideal solution fails to provide any performance improvement, ultimately converging to the Nash equilibrium.

#### Example 4

**Fig. 15:**
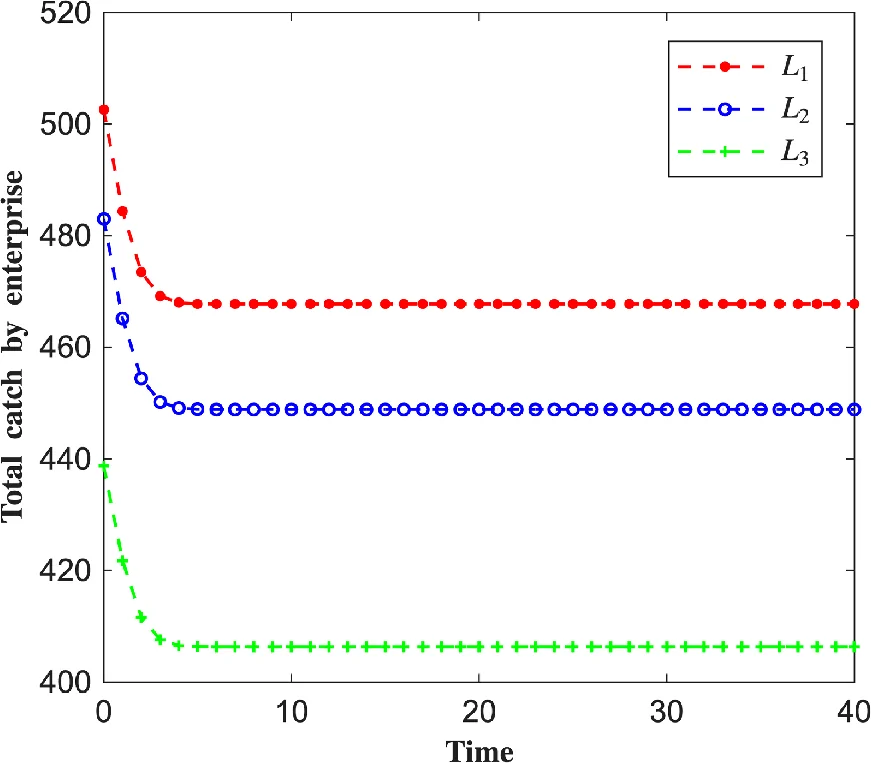
Total catch for each fisher based on the data from Example 4, where ${L}_{i}>0,$ for $i=\mathrm{1,2},3.$ Every fisher records a catch (Color figure online)

**Fig. 16:**
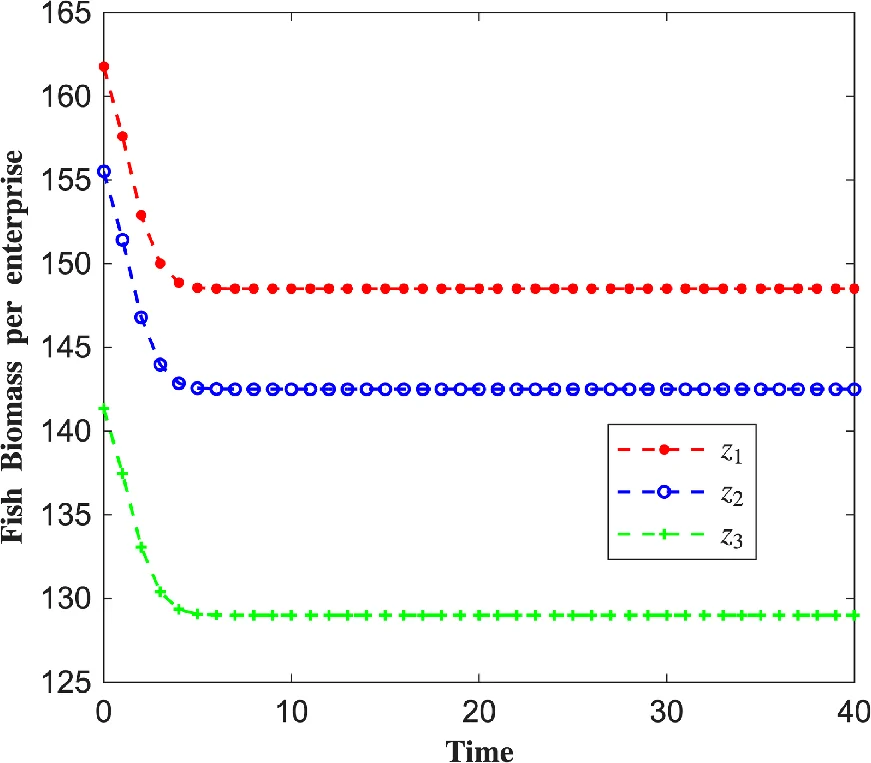
Evolution of fish dynamics (2) corresponding to Example 4, when $q\left({x}^{*,m}\right)\le p.$ (Color figure online)

**Fig. 17:**
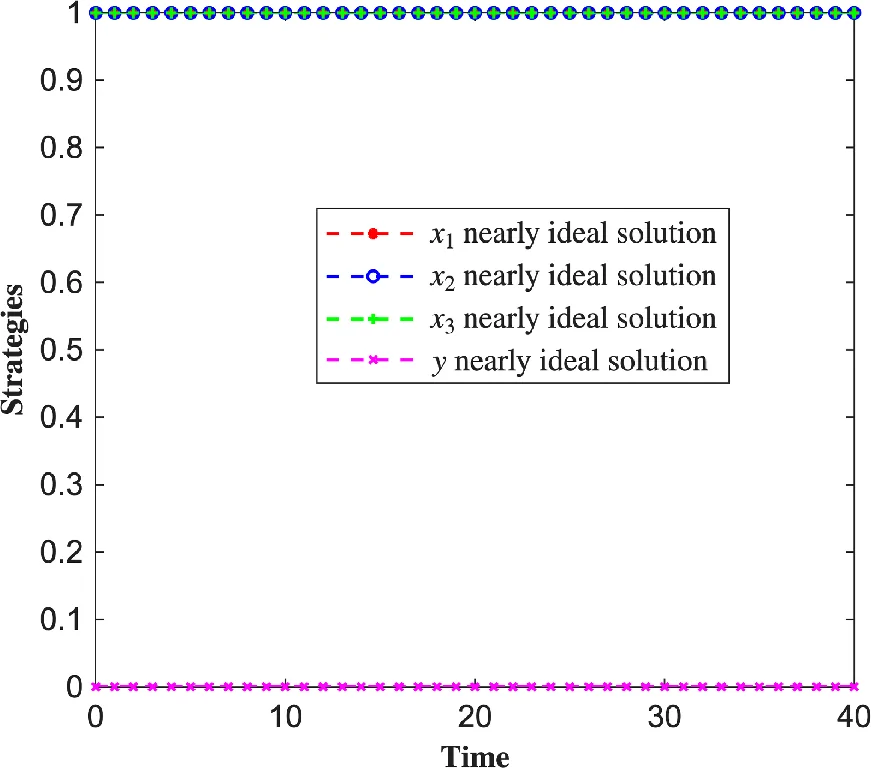
Strategy dynamics corresponding to Example 4, where all fishers sell their entire catch to the cooperative (Color figure online)

**Fig. 18:**
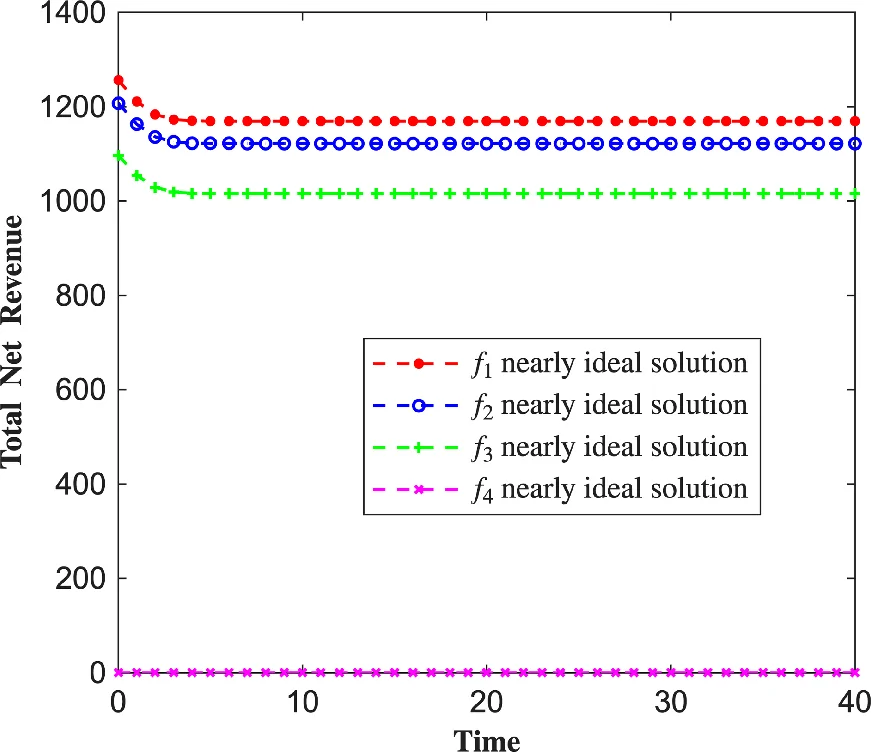
Total net revenue dynamics (11)–(12) for Example 4, regarding the fishers and cooperative, respectively. In this case, the cooperative’s benefit is zero (Color figure online)

**Fig. 19:**
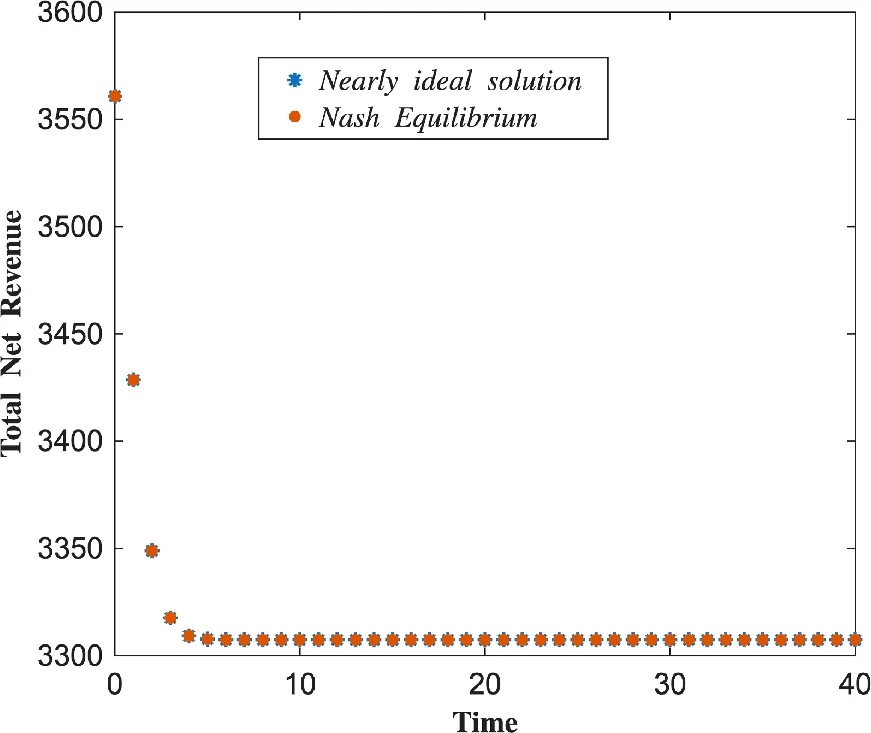
Comparison between solutions from game (13) and the Nash equilibrium corresponding to Example 4. The total net revenue of the nearly ideal solution simply matches that of the Nash equilibrium $\left(\mathrm{1,1}\right)$ (Color figure online)

An example where Theorem 2 does not hold occurs when $q\left({x}^{*,m}\right)\le p$. Figures 15, 16, 17, 18 and 19 illustrate this case for p=10, assuming β=1 and keeping the remaining parameters identical to those in Example 1. If condition (iii) of Theorem 2 is not satisfied, it logically follows that condition (ii) is also violated. Under this scenario, all fisher players' strategies converge to 1 as we can observe in Fig. 17, since selling the product outside the cooperative offers no extra benefit compared to doing so within the cooperative, in addition to the risk of the penalty this would entail. Therefore, all fisher players will choose to sell their entire catch to the cooperative. Then the cooperative's strategy drops to zero (Fig. 17), resulting in zero total revenue for the cooperative (Fig. 18). Consequently, the total net revenue of the nearly ideal solution simply matches that of the Nash equilibrium, offering no further performance improvement (see Fig. 19).

## Discussion and Outlook Concerning Similar Models

The game theoretical modelling of the conflict between a cooperative and its members, in evolutionary context, was first introduced in Varga (2010). It turned out that, based on a classical population dynamics with fishing effort, this game could be also extended to the case of fishery.

Competition in fisheries typically arises at two distinct stages: the resource-exploitation stage and the marketing stage. The present paper abstracts from the former by assuming that fishers operate independently in separate lakes, allowing us to focus exclusively on the latter. Specifically, we describe this marketing competition using a Cournot-type oligopoly model. This setting contrasts with classical fishery literature, which primarily focuses on competition for a common resource—widely known as the “tragedy of the commons” (see, e.g., Grafton et al. 2017). Although abstracting from the resource-exploitation stage via the independent lakes assumption is a simplification, it provides a clear framework to isolate and analyze marketing conflicts. Naturally, extending our study to incorporate both stages by modeling fishers competing in the same area remains a promising direction for future research.

In the present paper we have shown in what sense the proposed cooperative solution outperforms the known non-cooperative solution in the repeated game. The same issue would be also interesting for the case of a common resource.

The situation when there is a reserve area for the fish population could also be included, which would be a further development of Gámez et al. (2012). For possible extensions of the underlying Cournot-type oligopoly market model, we refer to Szidarovszky (2007), see also Baradaran (2019), which provide the theoretical basis to further analyze the asymptotic behavior of our solutions under competitive marketing dynamics.

Furthermore, a sequential timing of decisions can also be considered. For instance, a repeated dynamic Stackelberg game model for water rationalization in drought emergence, where the strategy choice depends on the previous strategy choices, was successfully applied in Kicsiny et al. (2014a). A similar approach could be applied in the context of our fishery model such that, according to the Stackelberg games, at every step, the cooperative would choose its strategy first, followed by the strategy choice of the members. The solution of such games would be technically rather involved (see e.g., Kicsiny et al. 2014b), so another paper should be dedicated to it.

Finally, for an outlook we also mention that the special cooperative solution called nearly ideal solution has already been applied to sustainable water use problems in Piscopo et al. (2024).

## Data Availability

In our simulations we worked only with illustrative parameter values specified in the paper, for our analysis we needed no experimental data.
